# Ultrasound to guided epicutaneo-caval catheter insertion in newborn infants

**DOI:** 10.3389/fped.2022.1022796

**Published:** 2022-11-17

**Authors:** Xiao-Ling Ren, Man Wang, Yu-Ru Wei, Jing Liu

**Affiliations:** ^1^ Department of Neonatology and NICU, Beijing Chao-Yang Hospital, Capital Medical University, Beijing, China; ^2^ Department of Neonatology and NICU, Beijing Chao-Yang District Maternal and Child Healthcare Hospital, Beijing, China

**Keywords:** epicutaneo-caval catheter, peripherally inserted central catheter, newborn infant, intensive care unit, tip navigation, tip location, radiography, ultrasound

## Abstract

**Objective:**

Recently, ultrasound (US) has been increasingly used for epicutaneo-caval catheter (ECC) tip positioning; however, the selection of blood vessels for ECC still depends on the operator’s subjective judgment. This study aimed to explore the value of US in decision-making regarding the great saphenous vein (GSV), tip navigation, and tip location of ECC.

**Methods:**

Catheterization through the GSV of the lower extremity was selected. The running condition of the GSV was assessed by using US, and the angle between the GSV and the femoral vein was observed and measured. We selected the GSV with a smaller angle to the femoral vein for ECC catheterization.

**Results:**

ECC catheterization under ultrasound guidance increased the success rate at the time of catheterization from 82.5% to 100% (increased by 17.5%) and shortened the catheterization time from 56.1 ± 5.30 min to 31.5 ± 2.58 min on average (shortened by 44%). The incidence rate of catheter-related complications decreased by 58.2% catheter days from 6.80/1,000 to 2.84/1,000.

**Conclusion:**

ECC insertion under the guidance of US has numerous advantages, including significantly improving the success rate of one-time catheterization, shortening the time of catheterization, and reducing catheter-related complications.

## Introduction

An epicutaneo-caval catheter (ECC) is one of the main intravenous routes used to infuse medications into critically ill newborns and premature infants (1, 2), and the technique and procedure are repeated many times almost every day in neonatal intensive care units (NICUs). Quick catheterization and accurate judgment of the position of the catheter tip are key to improving the success rate of catheterization. Chest x-ray (CXR) after ECC insertion in infants is the most commonly used method for verifying catheter tip position (1, 2).

However, x-ray positioning is associated with inevitable shortcomings. (1) During CXR positioning, babies need to keep their position fixed (usually supine) with their limbs in a straight state. However, crying among restless newborns easily leads to inaccurate positioning. (2) Both the clinical experience and literature indicate that even if the CXR shows the correct position of the ECC tip, it may still be inaccurate because the child usually moves, so the position of the limb changes during CXR positioning (2–4). (3) Inevitable radiation damage may occur, especially among developing premature infants and newborns, who are considerably more susceptible to ionizing radiation because the rate at which their cells undergo mitosis is more rapid than that observed in adult populations (5). Increased radiosensitivity, greater mitotic activity, and a protracted period for consequences to manifest lead to a two- to -threefold higher risk of radiation-induced cancer per unit of dose among preterm infants than among the average population (6). (4) CXR is consistently a postprocedural methodology since fluoroscopy is not considered appropriate in the NICU (4).

Therefore, the use of ultrasound (US) for catheter placement confirmation in the neonatal fields has been developed widely with a variety of advantages (7–10). Catheterization through the great saphenous veins (GSVs) of the lower extremities is the most commonly used approach for neonatal ECC (11, 12). However, the side of the GSV for catheterization is arbitrarily selected; thus, the success rate is also relatively low, and the procedure is time-consuming. In the present retrospective study, we explored the value of US in best GSV choice, tip navigation, and tip location of ECC.

## Patients, materials, and protocol

### Patients

The study was conducted in accordance with the Declaration of Helsinki and was approved by the Ethics Committee of Beijing Chao-Yang District Maternal and Child Health Care Hospital (No. 2011-LC-Ped-01). Written informed consent was obtained from the participants’ parents. Starting in January 2019, a total of 166 patients who underwent ECC through GSV were included in this study. They were divided into two groups: the US-guided group, which included 126 patients, and the non-US-guided group, which included 40 patients.

### Indications for ECC

The following conditions were indications for ECC in the present study: neonates need to be fed intravenously or treated intravenously for long periods, or they may need to be administered multiple medicines intravenously at the same time. These indications included extremely preterm birth and very low birth weight (45/166, 27.1%), respiratory distress syndrome (RDS) (37/176, 22.3%), severe sepsis or intrauterine infectious pneumonia (25/166, 15.1%), capillary leakage syndrome (CLS) (12/166, 7.2%), disseminated intravascular coagulation (DIC) (14/166, 8.4%), pulmonary hemorrhage (8/166, 4.8%), meconium aspiration syndrome (MAS) (7/166, 4.2%), persistent pulmonary hypertension of the newborn (PPHN) (6/166, 3.6%), severe asphyxia at birth (4/166, 2.4%), hyperinsulinemia (1/166, 0.6%), and other reasons (7/166, 4.2%).

### Materials

#### Ultrasound

A Voluson S10 (GE Healthcare, United States) Color Doppler ultrasound diagnostic system was used in this study.

#### ECC and needle

• Type of catheter: Medical grade single-lumen polyurethane.
• Catheter size: 1.9 Fr.
• Type of needle: 22G 304 stainless steel needle with a side hole and tear sheath.

### Routes of catheter placement

All patients were catheterized through the GSV route in this study.

### Protocol

#### Preprocedural evaluation

**Figure 1:**
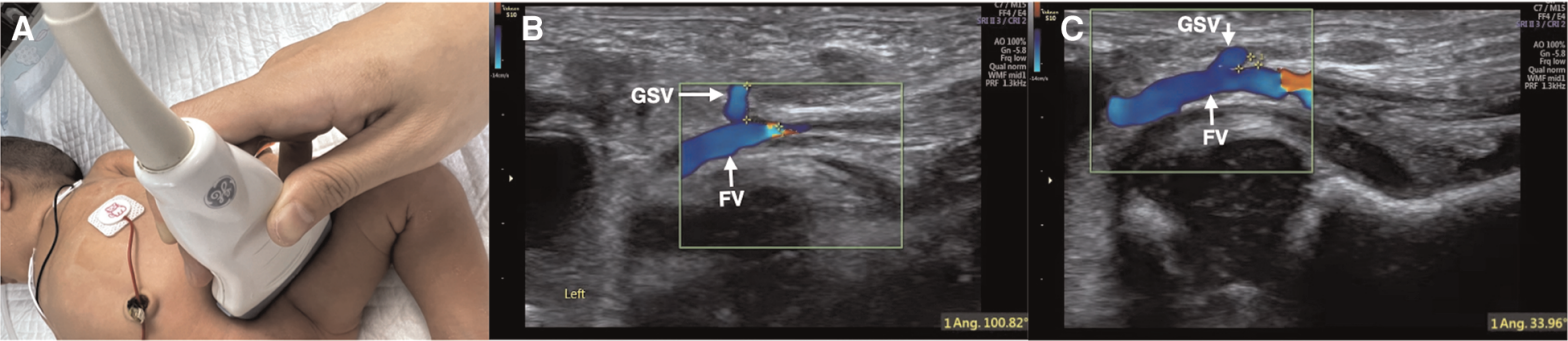
Color Doppler ultrasound measuring the angle between GSV and FV. Color Doppler ultrasound was used to assess the course conditions of bilateral GSV. The probe is placed in the groin (**A**). Measuring the angle between GSV and FV (**B**: angle = 100°; **C**: angle = 33.9°). The GSV on one side with a smaller angle between the two veins was selected for catheterization (**C**).

• **Probe:** Linear array probes [low-frequency probe 9L (3–8 MHz) or high-frequency matrix linear (ML) 6–15 (4–13 MHz)].
• **Selection of the GSV suitable for ECC insertion:**
  1. To obtain the wide axis view of GSV with GSV joining femoral vein (FV), the probe was placed on the groin area, and the wide axis of the probe was consistent with the course of the FV to obtain the wide axis view of GSV with GSV joining FV by using 2D US.
  2. To measure the angle between GSV and FV, the GSV was verified and highlighted using color Doppler ultrasound, and then the angle between the GSV and FV was then measured.
  3. The GSV on the side with a smaller GSV-FV angle was selected for ECC catheterization (Figure 1).
• **Selection of the insertion site**: The vessels on the medial knee joint or medial ankle joint were assessed, and one vessel that was easy to insert into the ECC was selected in the present study.

In the control group (non-US-guided group), the blood vessels and insertion sites were blinded and randomly selected.

• **Catheter secured:**
  1. To place the extravascular part of the catheter as U-, C- or S-shaped.
  2. To fix the catheter fixation wing to the skin using adhesive tape.
  3. To stabilize the whole *in vitro* portion of the ECC (Cover the fixation wing, U-, C-, or S-shaped catheters and insertion sites) using sterile transparent film.
  4. Finally, the catheter was cross-fixed with sterile adhesive tape.

#### Real-time evaluation

**Figure 2:**
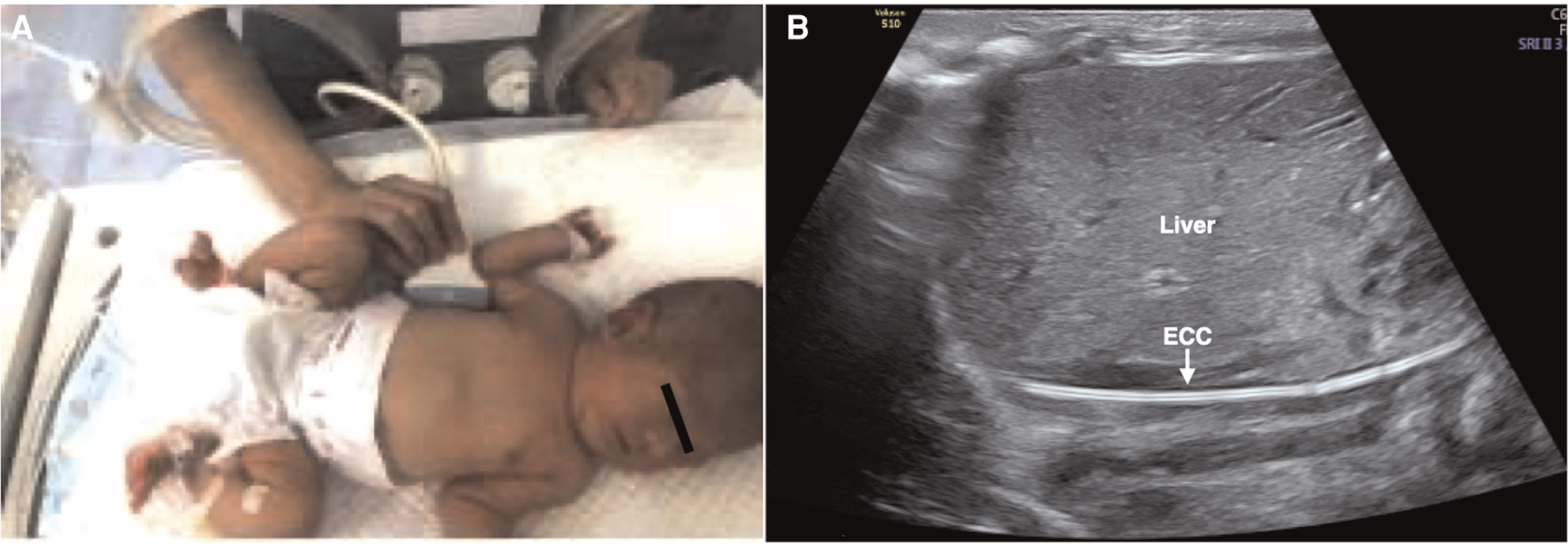
To determine whether the ECC catheter is located in the inferior vena cava. Right midaxillary line view. The probe was placed in the midaxillary line. The baby was placed in the supine position (**A**), and the ECC catheter showed a high echo “equal-like sign” (“=” line) appearance under ultrasound (**B**). There were no abnormal conditions, such as folding back of the catheterization (**B**).

• **Probe:** Linear array probes [low-frequency probe 9 L (3–8 MHz) with turning on convex (trapezoid) or micro convex probes C2–9 (frequency 2.5–9.1 MHz)].
• **To observe whether the ECC is located in the inferior vena cava (IVC):**
  1. The baby was placed in the supine position.
  2. In the right midaxillary line view, the probe was placed along the midaxillary line to obtain sections of the inferior vena cava with the parallel descending aorta. The upper boundary of the probe was about to flush with the 10th rib, and the rib was scanned vertically.
  3. If necessary, the probe could be slightly adjusted forward or backward along the narrow axis of the probe and sometimes deflected (approximately 5°–10°) to obtain a section of the inferior vena cava parallel to the descending aorta.
  4. The inserted catheter showed a high echo “equal-like sign” (“=” line) appearance under ultrasound (7). Therefore, we could observe whether the ECC catheter was located in the IVC and whether there are abnormal conditions, such as the catheter folding back (Figure 2).

#### Real-time tip location

**Figure 3:**
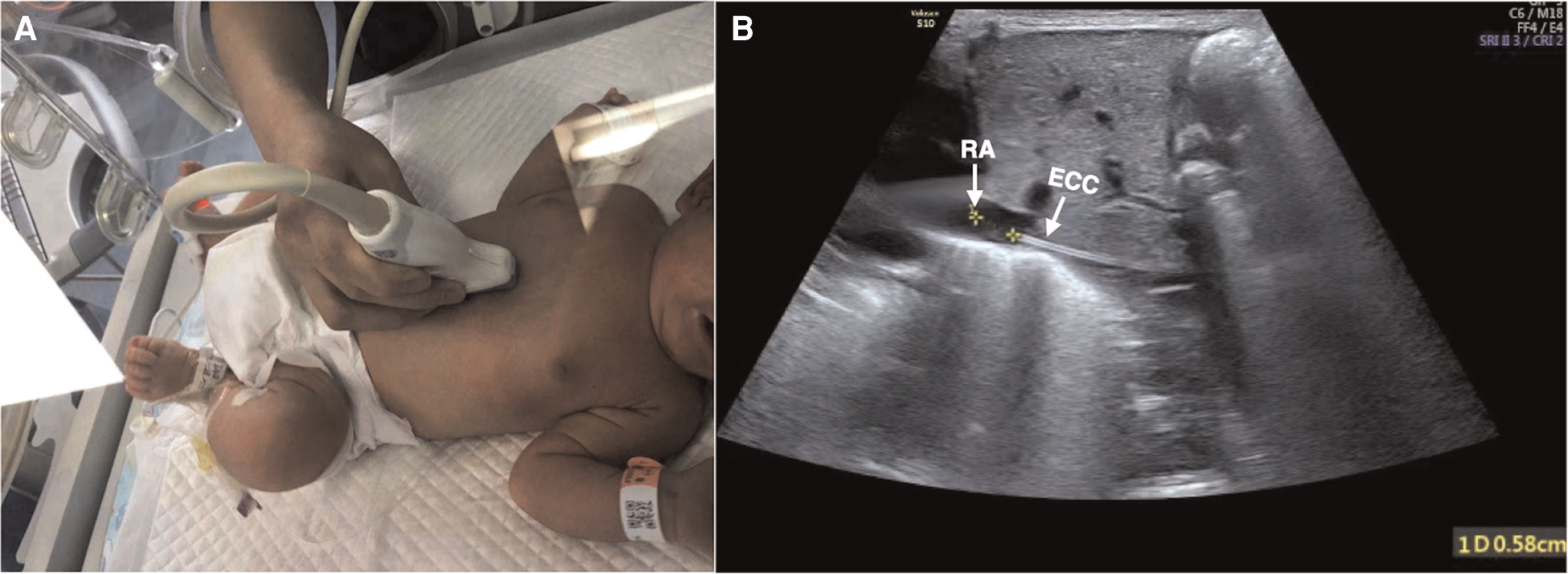
Tip location. View of the inferior vena cava draining into the right atrium. The baby was kept in the supine position, and the probe was placed obliquely to make the acoustic beam slightly deflect to the right and obtain the sagittal section of IVC (**A**). The correct placement of the catheter could be determined by measuring the distance between the catheter tip and the right atrium entrance, which was 0.58 cm in this infant (**B**).

• **Probe:** linear array probe 9L (frequency 3–8 MHz) with convex (trapezoid) turned on or microconvex probe C2–9 (frequency 2–10 MHz).
• **To measure the distance between the ECC tip and the right atrium entrance**:
  1. The baby was maintained in the supine position.
  2. Because of the inferior vena cava draining into the right atrium, the probe was moved under the xiphoid process and perpendicular to the chest wall, and the left lobe of the liver under the xiphoid process was the acoustic window.
  3. Then, the probe was slightly deflected to the right (5°–10°) to make the acoustic beam slightly deflect to the right of the baby and to then obtain the section of the inferior vena cava into the right atrium. Under real-time ultrasound, the correct placement of the catheter could be determined by measuring the distance between the catheter tip and the right atrium entrance (7). Generally, the optimal position was believed to be at the cavoatrial junction (1–3) (Figure 3).
  4. When necessary, a small flush of saline (0.5–1 ml) could be injected into the catheter to help identify the ECC tip (3, 7).

### Statistical analysis

The Statistical Package for the Social Sciences (SPSS) 24.0 software was used to statistically analyze the data. The time required for ECC tube placement was expressed as the mean ± standard deviation (*x¯* ± *s*).

The success rate of one-time catheterization was expressed as a percentage (%). The incidence rate of ECC complications was expressed as the numbers/1,000 catheter days. Student's *t*-test or the chi-square test was used to compare differences between the two groups. A value of *p* < 0.05 indicated statistically significant differences.

## Results

### Baseline characteristics

The baseline characteristics in the two groups are listed in Table 1, and the differences between preterm and full-term infants in the US-guided group are listed in Table 2.

**Table 1:** Baseline characteristics in two groups.

Groups	Premature/term	GA (weeks)	BW (kg)	Male/female	Age at insertion (days)	Duration of catheterization (day)	Complications of ECC (/1,000 catheter days)
US-guided group	91/35	33.1 ± 3.17 (27^+1^–41)	2.17 ± 2.71 (0.88–4.06)	77/49	2.70 ± 3.99 (0.5–26)	16.79 ± 10.15 (3–57)	2.84
Non-US-guided group	25/15	33.5 ± 3.20 (27–39^+6^)	2.10 ± 2.79 (0.90–4.00)	27/13	2.65 ± 4.02 (1.0–25)	18.37 ± 13.13 (7–58)	6.80
t	N/A	0.820	0.165	N/A	0.67	0.797	Decreased by 58.2%
*P*		0.815	0.264		0.66	0.699	

US, ultrasound; GA, gestational age; BW, birth weight; ECC, epicutaneo-caval catheter.

**Table 2:** The difference between preterm and full-term infants in ultrasound-guided group.

Groups	*N*	GA (weeks)	BW (kg)	Male/female	Age at insertion (day)	Duration of catheterization (day)	Reasons for ECC	Complications of ECC (/1,000 catheter days)
Preterm	110	32.3 ± 2.63	1.72 ± 0.50	68/42	2.72 ± 4.22	17.7 ± 10.4	Premature, LBW, RDS, DIC, PH, severe sepsis, etc.	3.08
Full-term	16	38.2 ± 1.15	3.28 ± 0.58	10/6	2.50 ± 1.74	10.6 ± 5.49	MAS, CLS, DIC, PPHN, asphyxia, etc.	0
*X*^2^/*t*	N/A	8.79	11.39	N/A	0.212	2.69	N/A	4.01
*P*		0.00	0.00		0.83	0.01		0.045

LBW, low birth weight; RDS, respiratory distress syndrome; DIC, disseminated intravascular coagulation; PH, pulmonary hemorrhage; MAS, meconium aspiration syndrome; CLS, capillary leakage syndrome; PPHN, persistent pulmonary hypertension of the newborn; ECC, epicutaneo-caval catheter; GA, gestational age; BW, birth weight.

### The success rate of one-time catheterization

In the US-guided group, all 126 patients had successful one-time catheterization (among them, there were 96 cases of medial GSV of the knee and 30 cases of GSV of the ankle). The success rate of one-time catheterization was 100%. In the 40 cases of the non-US-guided group, 33 cases were successful in one-time catheterization, and the success rate of one-time catheterization was 82.5%. The success rate of one-time catheterization under ultrasound guidance increased by 17.5% (*p* < 0.05) (Table 3).

**Table 3:** Success rate of one-time catheterization between two groups.

Groups	Patients	Success	Success rate (%)	Increased by (%)	*χ* ^2^	*p*
US-guided group	126	126	100	17.5	23.021	<0.001
Non-US-guided guided	40	33	82.5			

US, ultrasound.

### The time required for ECC placement

Among the 126 patients in the US-guided group, the average time required for ECC placement was 31.5 ± 2.58 min. In the 40 cases in the non-US-guided group, the average time required for ECC placement was 56.1 ± 5.30 min. Ultrasound guidance shortened the catheterization time of ECC by 44% (*t* = 46.84, *p* < 0.001) (Table 4).

**Table 4:** The time consumption between two groups.

Groups	Patients	Time consumption (min)	Shortened by (%)	*t*	*p*
US-guided group	126	31.5 ± 2.58	44.0	46.84	<0.001
Non-US-guided group	40	56.1 ± 5.30			

US, ultrasound.

### ECC-related complications and outcomes

The numbers of catheter-related complications/1,000 catheter days in the US-guided and non-US-guided groups were as follows: CLABSI (central line associated-blood stream infection) 1.89 vs. 2.72, thrombosis 0.473 vs. 0, occlusion 0.945 vs. 1.36, and secondary malposition 0 vs. 4.08. The results indicated that US-guided catheter insertion decreased the total incidence rate of ECC-related complications from 6.80/1,000 catheter days to 2.84/1,000 catheter days; that is, it reduced the total incidence rate of ECC-related complications by 58.2%. The incidence of ECC complications in preterm infants was significantly higher than that in term infants (Table 2). No pericardial effusion, cardiac tamponade, perforation, arrhythmia, or heart valve damage occurred in either group. All patients in the two groups recovered and were discharged.

## Discussion

As mentioned above, CXR has a number of disadvantages. To overcome these shortcomings, the ultrasound localization method has been gradually applied in clinical practice in recent years (3, 4, 7–10, 13–15), and studies or guidelines recommending ultrasound as the “gold standard” for determining the correct tip position have been developed (2–4).

Although ultrasound positioning is used and recommended for ECC tip positioning by experts and even the NeoECHOTIP and RaSuVa protocols (14, 16), the selection of blood vessels for ECC still depends on the operator's experience and subjective judgment, which still results in great blindness. Therefore, ECC tip positioning is subject to disadvantages, such as the low success rate of one-time insertion and the long time consumption of catheterization. Thus, a rapid catheterization method, which can improve the success rate of one-time catheterization and reduce discomfort during neonatal catheterization, needs to be explored. This is the first study that guided vessel selection and ECC tip positioning under real-time ultrasound monitoring. Generally, catheterization through the lower limb vein, that is, the GSV, is believed to be the preferred approach for neonatal ECC catheterization (11, 12). We first used two-dimensional ultrasound to find GSV and FV; then, we used color Doppler ultrasound to further identify the blood vessels suitable for placing ECC. Finally, we used ultrasound to locate the tip of ECC. Since color Doppler ultrasound can accurately measure the angle between GSV and FV, the use of an ultrasound monitoring method to guide the placement of ECC may have more advantages.

\Based on the obtained images of GSV and FV by two-dimensional ultrasound, we used color Doppler ultrasound to observe the direction of GSV, that is, the relationship between the GSV and FV, and measured the angle at which the GSV joined the FV. Then, the GSV on the side with the smaller angle between the GSV and FV was selected for ECC catheterization (Figure 1) to select the most suitable vessel for ECC placement. This selection is the key to improving the success rate, shortening the catheterization duration and reducing complications. According to the results of this study, the success rate of one-time catheterization increased by 17.5% from 82.5% to 100%. The average catheterization time was shortened from 56.1 ± 5.30 to 31.5 ± 2.58 min, which was shortened by 44% and reduced the total incidence rate of ECC-related complications by 58.2%. All patients in the two groups recovered and were discharged in this study.

Another important contribution of this study is the ECC tip navigation at the midaxillary line by using ultrasound. The advantages of observing ECC in this position are as follows. (1) Almost the whole course of the inferior vena cava can be seen, and the position relationship between ECC and the inferior vena cava can be clearly found. (2) During catheterization, complications such as catheter displacement or disconnection can be easily detected and corrected in time. (3) The catheter tip position was easy to locate. Because this method is easy to learn and master, it is suitable for widespread application in NICUs.

Although ECCs are often called peripherally inserted central catheters (PICCs) because they actually insert central venous access devices through peripheral, superficial veins (1, 2), they are very different (2). ECCs are small-bore catheters (1–2.7 Fr) made of silicone or old-generation polyurethane inserted *via* superficial veins of the limbs or scalp using direct vein visualization. PICCs are larger catheters (3.0 Fr and larger) made of new-generation polyurethane, usually power-injectable, inserted into the deep veins of the arm (brachial, basilic, axillary) using ultrasound guidance. In this study, the GSV that we selected for catheter placement was a superficial peripheral vein, and the size of the catheter was small (1.9 Fr). Therefore, this catheterization should be classified as ECC rather than PICC based on the above differences.

The ECC used in our NICU were nonantimicrobial-treated catheters. Although a new type of antimicrobial ECC coated with antibiotics and antifungals is available on the market, controlled studies on its effectiveness in reducing catheter-related bloodstream infections are lacking (2). We believe that strict disinfection care at the puncture site is the key to avoiding and reducing ECC-related infections. Dynamic monitoring of catheter status by ultrasound during the application of ECC is an important method for the early detection and prevention of thrombosis, malpositions, or occlusions.

In conclusion, as an intraprocedural methodology, ultrasound is believed to be the most promising tool for tip navigation and location during the placement of central venous access devices in neonates, and it is appropriate for both tip navigation and tip location (3). The present study also proved that ECC catheterization under ultrasound guidance has outstanding advantages, significantly improving the success rate of one-time catheterization and shortening the time of catheterization and significantly decreasing catheter-related complications with no radiation damage, which warrants wide application in clinical practice (2, 3, 10, 14).

## Data Availability

The original contributions presented in the study are included in the article/Supplementary Material, further inquiries can be directed to the corresponding author.
